# Treatment with the PARP inhibitor, niraparib, sensitizes colorectal cancer cell lines to irinotecan regardless of MSI/MSS status

**DOI:** 10.1186/s12935-015-0162-8

**Published:** 2015-02-04

**Authors:** Sybil M Genther Williams, Apryle M Kuznicki, Paula Andrade, Brian M Dolinski, Cem Elbi, Ronan C O’Hagan, Carlo Toniatti

**Affiliations:** Department of Oncology, Merck Research Laboratories, 33 Avenue Louis Pasteur, Boston, MA 02115 USA; Department of In Vivo Pharmacology, Merck Research Laboratories, 33 Avenue Louis Pasteur, Boston, MA 02115 USA; Current address: Bayer HealthCare, 100 Bayer Road, Whippany, NJ 07891 USA; Current address: Institute for Applied Cancer Science, 1901 East Road, Unit 1956, Room 4SCR6.1009, Houston, TX 77005 USA

## Abstract

**Background:**

Cells with homologous recombination (HR) deficiency, most notably caused by mutations in the *BRCA1* or *BRCA2* genes, are sensitive to PARP inhibition. Microsatellite instability (MSI) accounts for 10-15% of colorectal cancer (CRC) and is hypothesized to lead to HR defects due to altered expression of Mre11, a protein required for double strand break (DSB) repair. Indeed, others have reported that PARP inhibition is efficacious in MSI CRC.

**Methods:**

Here we examine the response to niraparib, a potent PARP-1/PARP-2 inhibitor currently under clinical evaluation, in MSI versus microsatellite stable (MSS) CRC cell lines *in vitro* and *in vivo*. We compiled a large panel of MSI and MSS CRC cell lines and evaluated the anti-proliferative activity of niraparib. In addition to testing single agent cytotoxic activity of niraparib, we also tested irinotecan (or SN-38, the active metabolite of irinotecan) activity alone and in combination with niraparib *in vitro* and *in vivo.*

**Results:**

In contrast to earlier reports, MSI CRC cell lines were not more sensitive to niraparib than MSS CRC cell lines*¸* suggesting that the MSI phenotype does not sensitize CRC cell lines to PARP inhibition. Moreover, even the most sensitive MSI cell lines had niraparib EC50s greater than 10 fold higher than BRCA-deficient cell lines. However, MSI lines were more sensitive to SN-38 than MSS lines, consistent with previous findings. We have also demonstrated that combination of niraparib and irinotecan was more efficacious than either agent alone in both MSI and MSS cell lines both *in vitro and in vivo*, and that niraparib potentiates the effect of irinotecan regardless of MSI status.

**Conclusions:**

Our results support the clinical evaluation of this combination in all CRC patients, regardless of MSI status.

## Introduction

Poly (ADP-ribose) polymerase (PARP) enzymes are involved in repair of single strand DNA lesions using the base excision repair (BER) pathway. Inhibition of PARP enzymes induces persistence of single strand breaks (SSBs) which can cause double strand breaks (DSBs) when the SSBs are encountered by a replication fork. The development of PARP inhibitors as agents to treat cancers with homologous recombination (HR) defects is based on the idea that cells with defects in DSB repair, such as BRCA-deficient cells, are more dependent on PARP and BER to maintain genomic integrity [1,2]. Indeed, preclinical and clinical evidence have demonstrated that PARP inhibitors are synthetic lethal for tumors with mutations in the *BRCA1* and *BRCA2* genes and other genes involved in HR [1-5].

The instability of microsatellite repeated sequences, MSI, is found in tumors from the familial cancer syndrome hereditary nonpolyposis colorectal cancer (HNPCC) and in 10-15% of sporadic CRC. The MSI phenotype is a marker of an underlying mismatch repair (MMR) defect which stems from germline mutation in one of the MMR genes (principally *MLH1* or *MSH2*) or aberrant methylation of the *MLH1* promoter. One consequence of MSI is the reduced expression of the Mre11 protein resulting from mutation of the poly(T) 11 repeat within intron 4 of human *MRE11* [6]. Reduced expression of Mre11 is hypothesized to lead to defects in HR, due to Mre11’s essential role in sensing DSBs and facilitating their repair [6-10].

PARP inhibition is effective in combination with irradiation and DNA-damaging agents [11]. In particular, PARP inhibitors have been shown to potentiate the effects of Topoisomerase 1 (Top 1) inhibitors both preclinically and clinically [12-17]. Top1 inhibition slows replication fork progression and induces the widespread formation of unusual replication intermediates, most notably reverse replication forks [12]. PARP activity is required for effective fork reversal, which limits the number of DSBs that result [12].

Irinotecan, a Top 1 inhibitor, is used as a therapy for CRC either alone or in combination with leucovorin and 5-Fluorouracil (5-FU). MSI is associated with increased sensitivity to irinotecan, both *in vitro* and in patients with advanced colon cancer [9,18,19]. The mechanism underlying this observation is not well understood.

Niraparib is a potent and selective orally available PARP-1/2 inhibitor [3]. *In vitro* and *in vivo*, niraparib displays outstanding monotherapy efficacy in a large panel of BRCA mutant cell lines with at least 10-fold selectivity over BRCA wild type cell lines [3]. In this study, the efficacy of niraparib was evaluated in the presence and absence of irinotecan in models of CRC with defects associated with the MSI phenotype as compared to MSS phenotype.

We demonstrate that the MSI phenotype does not overtly sensitize CRC cell lines to PARP inhibition and confirm that MSI CRC cell lines are more sensitive to SN-38 (active metabolite of irinotecan) than MSS cell lines. Niraparib potentiated the cytotoxic activity of irinotecan in both MSI and MSS CRC models. Our data suggests that both MSI and MSS patient populations will benefit from the combination of niraparib and irinotecan.

## Methods

### Microsatellite repeat analysis

DNA was extracted using standard methods from cells that were plated one day prior. Cell lines that were used in this study were described as being either MSI or MSS in previous publications [20-25]. The cell lines analyzed for MSI included: **MSI**- COLO205, DLD-1, HCT8, HCT15, HCT116, LOVO, LS411N, RKO, RKO-E6, SW48 and **MSS**-SW403, SW1417, WIDR. Primers used to amplify BAT-25 and BAT-26 were (BAT-25) 5′- 6FAM-TCG CCT CCA AGA ATG TAA GT-3′ and 5′-TCT GCA TTT TAA CTA TGG CTC-3′ (BAT-26) 5′-HEX- TGA CTA CTT TTG ACT TCA GCC-3′ and 5′-AAC CAT TCA ACA TTT TTA ACC-3′. PCR amplification was performed with primers at 200nM each with 1X concentration of colorless GoTaq Flexi buffer (Promega Cat. No. M8305), 2 mM MgCl2, 0.2 mM of each dNTP (PCR nucleotide Mix Promega cat. No. C1141), 1.25 u of GoTaq DNA polymerase (5 u/μl; Promega cat. No. M8305), 50 ng of DNA, and nuclease free water to 50 μl. PCR conditions were: 95°C for 2 minutes followed by 35 cycles of 94°C for 1 minute, 55°C for 1 minute and 72°C for 1 minute, followed by a final extension of 72°C for 5 minutes and the 4°C. To analyze the PCR products the following mix was prepared: 10 μl Hi-Dri Formamide (Applied Biosystems Cat. No. 4311320) + 0.05 μl GeneScan 500 LIZ marker (Applied Biosystems Cat. No. 4322362). PCR product was diluted 1:400 in water. 1 μl of PCR product was added to 10 μl of Hi-Dri Formamide/Gene Scan 500 LIZ marker mix and samples were heated at 95°C for 5 minutes. The fluorescently labeled PCR products were detected using the Sequencer 3730 DNA analyzer (Applied Biosystems) and analyzed using Peak Scanner software (Applied Biosystems).

### Western analysis

10 cm dishes were lysed with 50–150 μl (depending on cell density) of boiling 1% SDS Lysis Buffer [50 ml-10% SDS (5 ml) 5 M NaCl (1 ml), 1 M Tris pH 7.5 (500 μl) dH20 (43.5 ml)] and put at 95°C for 5 minutes. Protein concentration was assessed in a 96 well format by BCA Protein assay Kit (PIERCE Catalog number: 23225). 30 μg of protein extract was loaded onto 10% Tris-Glycine SDS-PAGE gels and run at 100 Volts for 90 minutes. The gels were transferred onto PVDF membrane and incubated overnight at 4°C with primary antibody,1:1000 rabbit Anti-MRE11 Antibody (NOVUS Biologicals Catalog number: NB100-276 diluted in 5% non fat dry milk/TBS-T. The secondary antibody used was a 1:5000 dilution of ECL Anti-rabbit IgG Horseradish Peroxidase-Linked Species specific F(ab’)2 Fragment (donkey) (Amersham Catalog number: NA9340). Membranes were incubated for 1 hour in secondary antibody and developed using Pierce Supersignal West Dura Extended Duration Substrate (Catalog number: 34705).

### Proliferation assays

All of the cell lines were obtained from ATCC (Manassas, VA). **MSI-** DLD-1, HCT-8, HCT-15, HCT116, LOVO, LS174T, LS180, LS411N, RKO, RKOE6, SNUC2A, SW48. **MSS-** COLO205, HT29, NCI-H-508, SK-CO-1, SW403, SW480, SW620, SW837, SW948, SW1116, SW1417, SW1463, T84. To carry out 7 day monotherapy proliferation assays with the cell lines, 500–32,000 cells (cell line-dependent) were seeded in 96-well clear tissue culture plates (190 μl/well) in an appropriate tissue culture medium supplemented with FBS. The plate was incubated for 4 hours at 37°C, and niraparib was added in a 9 point titration, 3-fold dilutions starting at 10 μM for niraparib and starting at either 10 μM, 1 μM, or 100nM for SN-38 (in 9 point titration, 3-fold dilutions). The cells were then incubated for 7 days at 37°C, 5% CO_2_ (except cell lines grown in L-15 medium which were grown in at 37°C, 0% CO_2,_ 100% air) and the cell viability was assessed by WST-1 assay (Roche) as described by the Manufacturer. To carry out 7 day combination proliferation assays with the cell lines, 500–32,000 cells (cell line-dependent) were seeded in 96-well clear tissue culture plates (180 μl/well) in an appropriate tissue culture medium supplemented with FBS. The plate was incubated for 4 hours at 37°C, and niraparib was added at 125 nM, 250 nM, or 1 μM and SN-38 was added in an 8 point titration, 3-fold dilutions, starting at 100 nM. The cells were then incubated for 7 days at 37°C, 5% CO_2_ (except for the cell lines grown in L-15 medium which were grown in at 37°C, 0% CO_2,_ 100% air) and the cell viability was assessed by WST-1 assay (Roche) as described by the Manufacturer. The number of living cells was determined by reading the plate at 450 nm on a spectrophotometer. The signal produced is directly proportional to the cell number as the cells convert tetrazolium salt due into a formazan end product. Each experiment was run in duplicate. Cell growth was expressed as the percentage growth with respect to vehicle treated cells. The concentration required to inhibit cell growth by 50% (EC_50_) was determined using the four-parameter fit in SoftMax Pro 5.2. The Wilcoxon rank sum test was performed to determine statistical significance**.** Excess Volume HSA combination index was calculated using a MATLAB algorithm as described previously [26].

### *In vivo* xenograft studies

6 week old CD1 nu/nu mice were injected subcutaneously with either 5X10^6^ HT29 cells in 50% matrigel or 5X10^6^ HCT116 cells in 50% matrigel. When the average tumor size reached to 150 mm^3^ for HT29 and HCT116, mice were randomized to form homogeneous groups, and treatment started. Tumor measurements were recorded bi-weekly throughout the course of each study. Animals were dosed orally (p.o.) with 50, 25, or 10 mg/kg (mpk) niraparib (5 ml/kg in 0.5% w/v methylcellulose) each day for 3, 5, or 7 days (according to individual study design) alone or in combination with 100 mpk irinotecan (10 ml/kg) dosed intraperitoneally (ip.), once per week (qwk.), on day 3 for 4 weeks depending on treatment group. For tumor relapse studies, animals were treated for 4 weeks as described above, and then treatments were withdrawn and tumor relapse was monitored until the average tumor volume for each group reached 1000 mm^3^. Each animal study was conducted with 7–10 mice per individual treatment group. All animal studies were conducted in a specific pathogen-free environment in accordance with the internal Institutional Animal Care and Use Committee (IACUC) and other relevant standards.

## Results

**Figure 1:**
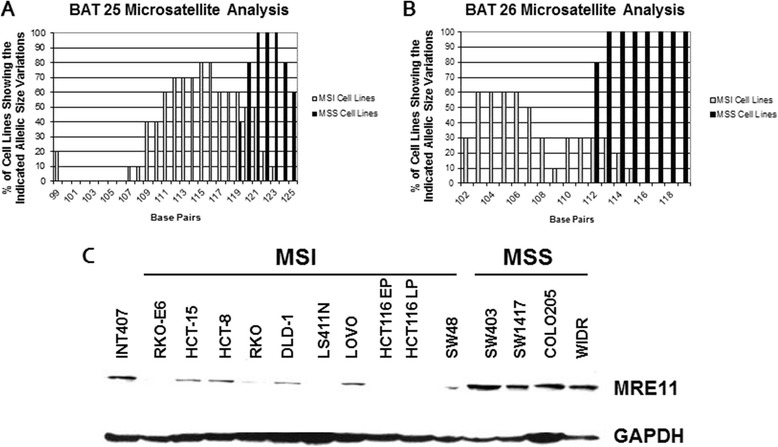
**Confirmation of MSI/MSS status of cell lines and MRE11 protein analysis. A** and **B**. We performed PCR using fluorescently labeled primers specific to BAT-25 **(A**) or BAT-26 **(B)** and then performed fragment analysis using Peak Scanner Software (Applied Biosystems). We observe that MSS cell lines (black) are more consistent with regard to size an allelic variation than the MSI cell lines (grey) and confirm the MSI/MSS status previously reported for these cell lines. **(C)** Western Analysis of MSI and MSS CRC cell lines confirms that Mre11 levels are reduced or absent in MSI cell lines, but are normal in MSS cell lines. Lysates from the normal immortalized colon epithelial cell line, INT407, serves as the normal tissue control. EP and LP refer to early or late passage of HCT116 cells.

BAT-25 is a poly (**T**) tract intragenic to the c-kit proto-oncogene assigned to 4q12 and BAT-26 is a poly **(A)** tract located in the 5^th^ intron of the hMSH2 gene. The BAT-25 and BAT-26 mononucleotide repeats are reported to be quasi-monomorphic meaning that there is not a significant size variation either between the alleles of one individual or between alleles of different individuals. This property permits the easy identification of MSI status without the use of a normal tissue control or on cell lines. MSI/MSS status across a CRC cell line panel was determined via PCR fragment analysis of the BAT-25 and BAT-26 mononucleotide repeats. MSS cell lines had longer and more uniform BAT-25 and BAT-26 alleles than MSI lines on average (Figure 1A and B). This data confirmed that the cell lines used in our cell line panel were MSI or MSS, as previously reported [20-25].

Cells with MSI are hypothesized to be HR-deficient due to reduced expression of Mre11 and subsequently the reduced expression of the Mre11-Nbs1-Rad50 complex. This reduction in expression has been shown to result from a mutation in the poly(T) 11 repeat within the human *MRE11* intron 4 [6]. Western analysis confirmed reduced Mre11 protein levels in MSI as compared to MSS cell lines (Figure 1C), consistent with previous reports [6,7,10,27].

**Figure 2:**
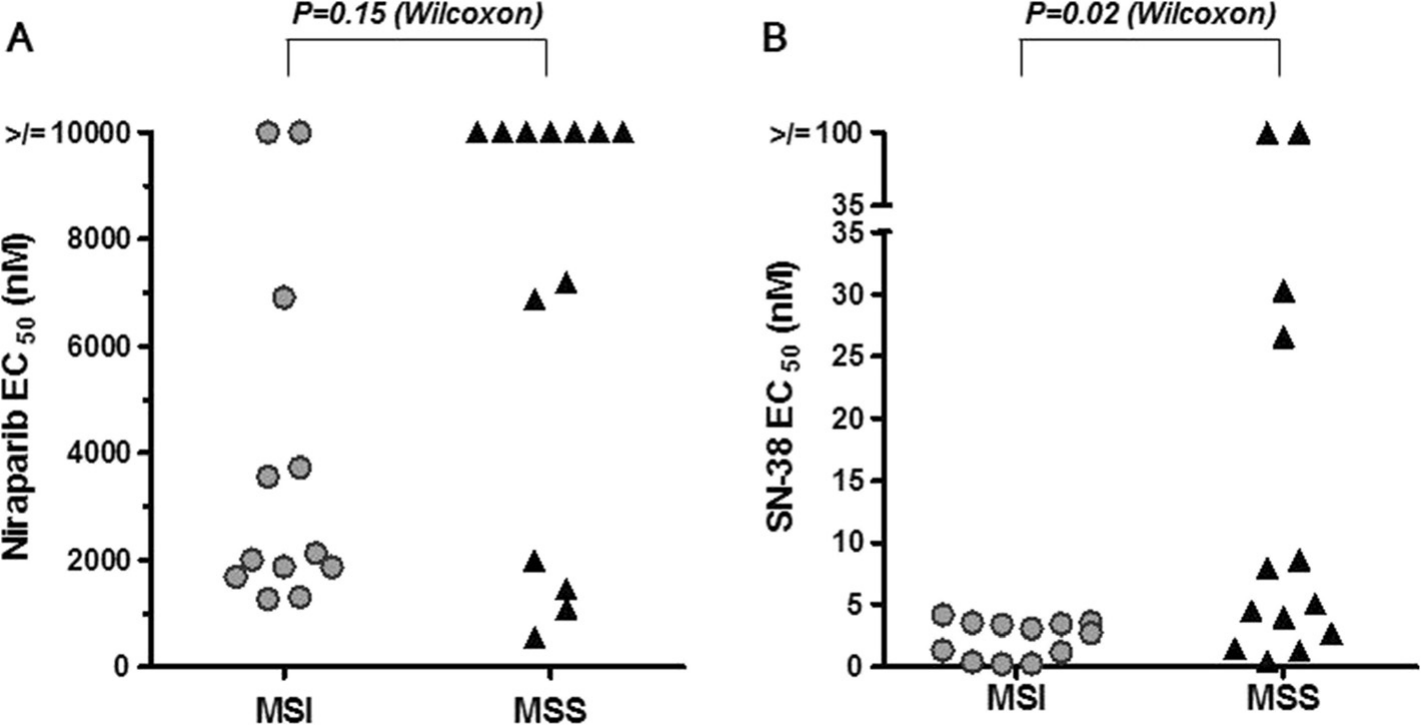
**MSI cell lines do not respond better to niraparib monotherapy than MSS cell lines, but do respond better to SN-38 than MSS lines. A**. MSI cell lines are not significantly more sensitive to niraparib than MSS cell lines. Niraparib EC50 values from 7 day proliferation assays in 12 MSI and 13 MSS CRC cancer cell lines. Each symbol indicates the EC50 of a single cell line and results are representative of 2 independent experiments. Cell lines that did not respond to niraparib within the tested dose range (10000 nM and below) are shown at 10000 nM. **B**. MSI cell lines are more sensitive to SN-38 treatment than MSS cell lines. Niraparib EC50 values from 7 day proliferation assays in 12 MSI and 13 MSS CRC cancer cell lines. Each symbol indicates the EC50 of a single cell line and results are representative of 2 independent experiments. Cell lines that did not respond to niraparib within the tested dose range (100 nM and below) are shown at 100 nM. The Wilcoxon rank sum test was performed to determine statistical significance.

Enhanced sensitivity to PARP inhibition was postulated for MSI CRC cell lines due to the reduction in Mre11 protein expression. To test this hypothesis, the cell line panel was expanded (12 MSI and 13 MSS) and 7-day proliferation assays were performed with niraparib. Although MSI cell lines on average did have lower 7 day proliferation EC50 values (Ave = 1823 nM) than MSS cell lines (Ave = 6859 nM), this difference was not statistically significant (p = 0.15 Wilcoxon Rank Sum Test; Figure 2A and Table 1), in contrast to what has been previously reported [28,29]. A semi-quantitative assessment of the amount of Mre11 expression relative to GAPDH expression was also performed, and there was no correlation between the level of Mre11 expression and sensitivity to niraparib (data not shown).

**Table 1:** **Niraparib potentiates the effects of SN-38 in MSI and MSS cell lines**

		**7 day EC50 (nM)**		**Combination EC50 (shift of SN-38 EC50 in presence of Niraparib)**		
				**Niraparib**		
	**Cell line**	**Niraparib**	**SN-38**	**125 nM**	**250 nM**	**1000 nM**
**MSI**	DLD1	3560	3.63	0.992	1.67	1.68
	HCT8	2130	3.60	2.07	1.54	1.35
	HCT15	1685	3.77	3.47	3.44	1.73
	HCT116	6915	0.180	0.125	0.155	0.0991
	LOVO	2010	3.43	2.3	3.53	2.4
	LS174T	1310	0.331	0.257	0.208	0.231
	LS180	3730	0.474	0.399	0.395	0.389
	LS411N	1875	3.57	1.21	1.11	0.729
	RKO	1870	0.958	0.593	0.778	0.184
	RKOE6	1270	0.730	0.426	0.422	0.464
	SNUC2A	>10000	3.23	3.04	2.72	2.52
	SW48	>10000	1.79	0.886	0.762	0.598
**MSS**	COLO205	1990	1.57	0.572	0.475	0.531
	HT29	6880	4.29	3.07	3.64	1.75
	NCI-H-508	>10000	3.63	1.70	3.31	1.41
	SK-CO-1	1090	0.416	0.178	0.334	0.143
	SW403	>10000	3.39	1.14	1.87	0.387
	SW480	>10000	29.0	11.9	11.6	8.7
	SW620	552	5.91	3.62	UR	UR
	SW837	>10000	UR	UR	UR	UR
	SW948	7195	6.67	3.85	4.47	2.80
	SW1116	>10000	UR	UR	UR	UR
	SW1417	>10000	27.7	29.2	28.3	31.0
	SW1463	>10000	26.4	22.2	UR	UR
	T84	1460	1.01	0.491	0.352	0.210

7 day EC50 data for niraparib alone, SN-38 alone, and the combination EC50 at 125, 250 and 1000 nM of niraparib in a panel of 25 MSI and MSS CRC cell lines . The niraparib EC50 values are the average of n = 2 from previous experiments. The SN-38 EC50 values reported are for n = 1 for the experiment done on that day. Values listed under 125, 250, and 1000nM niraparib are the EC50 values for SN-38 in the presence of 125, 250 or 1000 nM niraparib (combination EC50). EC50 data was calculated using the inflection point of the curve in a 4 parameter fit in SoftMax Pro 5.2. UR indicates that the data is unreportable due to an inadequate curve.

Proliferation assays were also performed on the panel of cell lines with SN-38. In agreement with previous reports, MSI CRC cell lines were significantly more sensitive to SN-38 monotherapy than MSS cells (p = 0.02 Wilcoxon Rank Sum Test; Figure 2B and Table 1) [18]. All of the MSI cell lines were sensitive to SN-38 with EC50s less than 5 nM, and although on average the MSS cell lines were less sensitive, 6/13 cell lines did have EC50s less than 5 nM.

**Figure 3:**
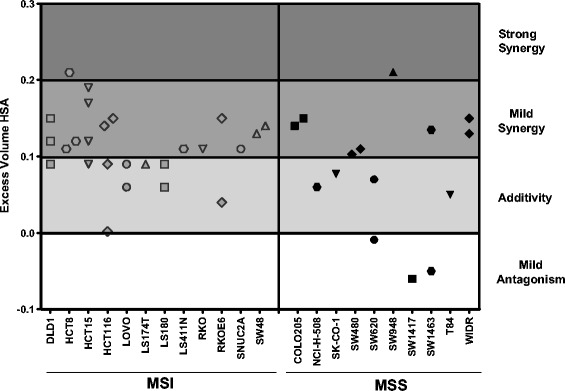
**Niraparib potentiates the effects of SN-38 in MSI and MSS cell lines.** Assessment of synergy with niraparib and SN-38 by excess volume HSA. Each symbol indicates a single experiment with the indicated cell lines. Data was generated from 7 day proliferation assay and excess volume HSA was calculated using a MATLAB algorithm. HSA values from 0.2-0.3 indicate strong synergy, 0.1-0.2 indicated mild synergy, 0.0-0.1 indicate additivity, and negative numbers indicate mild antagonism. Data points are from 1–3 experiments and are only shown if the p value associated with the excess volume HSA is equal to or less than 0.05.

PARP inhibitors have been shown to potentiate the effects of Top1 inhibitors both preclinically and clinically [12-17]. In order to test if niraparib potentiates irinotecan in MSI and MSS CRC cell lines, *in vitro* combination studies with niraparib (125 nm, 250 nM, and 1000 nM) and SN-38 (8 point dose response) were performed. As expected, we observed a shift of the SN-38 EC50 an average of 2-fold lower when niraparib was tested in combination with SN-38 as compared to when SN-38 was tested alone in both MSI and MSS CRC cell lines (Table 1). This data demonstrates that if a cell line responds to SN-38 monotherapy *in vitro*, niraparib potentiates that effect, regardless of MSI/MSS status. In addition, when Highest Single Agent (HSA) combinatorial analysis was applied to this data, we observe that combination of niraparib with SN-38 results in additive to synergistic inhibition of cell proliferation regardless of MSI/MSS status *in vitro* (Figure 3).

**Figure 4:**
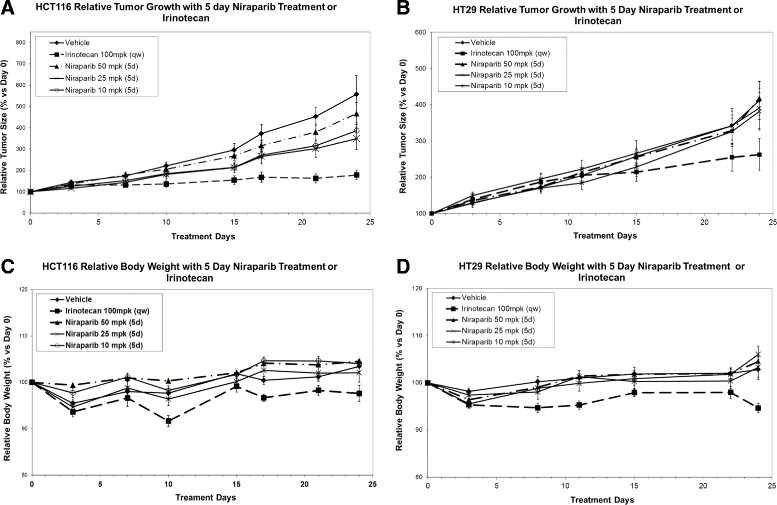
**Niraparib monotherapy is not as efficacious as irinotecan monotherapy in the HCT116 (MSI) or HT29 (MSS) xenograft models. A**. and **B**. *In vivo* efficacy of niraparib monotherapy on the HCT116 (MSI) and HT29 (MSS) xenograft models. Niraparib was dosed at 50, 25, or 10 mpk p.o. q.d., for 5 days a week (2 days off) for 4 weeks. Relative tumor size (% versus Day 0) is shown with SEM in error bars. **C**. and **D**. The 5 day dosing regimen was well tolerated with less than an average of 10% body weight loss in all doses tested. Relative body weight (% versus Day 0) is shown. For each group, n = 7-10.

In order to extend these observations, xenograft studies employing MSI (HCT116) or MSS (HT29) models were performed. HCT116 (MSI) or HT29 (MSS) xenograft tumor-bearing mice were dosed with niraparib at 10, 25, or 50 mpk (oral, daily) for 5 days per cycle (4 cycles) and efficacy at these doses was compared to irinotecan monotherapy (100 mpk, ip., qwk; 4 cycles). In both models, irinotecan monotherapy was more efficacious than niraparib monotherapy at the niraparib doses tested (Figure 4). In HCT116, the average tumor volume at day 28 for the irinotecan treated group was 225 mm^3^ and the tumor volumes for the niraparib single agent treated groups were 605, 734 and 739 mm^3^ for 10 mpk, 25 mpk and 50 mpk niraparib treated groups, respectively. When compared to vehicle control, only the average tumor volume of the irinotecan treated group was statistically different than the average of vehicle control (p = 0.0002 one-tailed homoscedastic student’s T test). In HT29, the average tumor volume for the irinotecan treated group at day 24 was 542 mm^3^ whereas the tumor volumes for the niraparib treated groups were 934, 802 and 768 mm^3^, for the 10 mpk, 25mpk and 50 mpk, niraparib treated groups, respectively. (Tumor volumes at day 24 were recorded for HT29 as compared to day 28 for HCT116 due to the day when vehicle control treated groups reached the maximum tumor volume of 1000 mm^3^.) In the HT29 model, none of the single agent groups (including irinotecan) had average tumor volumes that were statistically different as compared to vehicle control (irinotecan versus vehicle control p value = 0.06 one-tailed homoscedastic student’s T test). Niraparib was dosed no higher than 50 mpk in these studies because higher concentrations of niraparib were not tolerated in combination with irinotecan dosed at 100 mpk, qwk (data not shown). The maximum tolerated dose for niraparib as a single agent in mice is 100 mpk daily (data not shown). Doses of niraparib at 50 mpk (daily) gives approximately the same C min values as 40 mg/day in humans, the lowest dose that demonstrated clinical benefit (stable disease) in humans [30].

**Figure 5:**
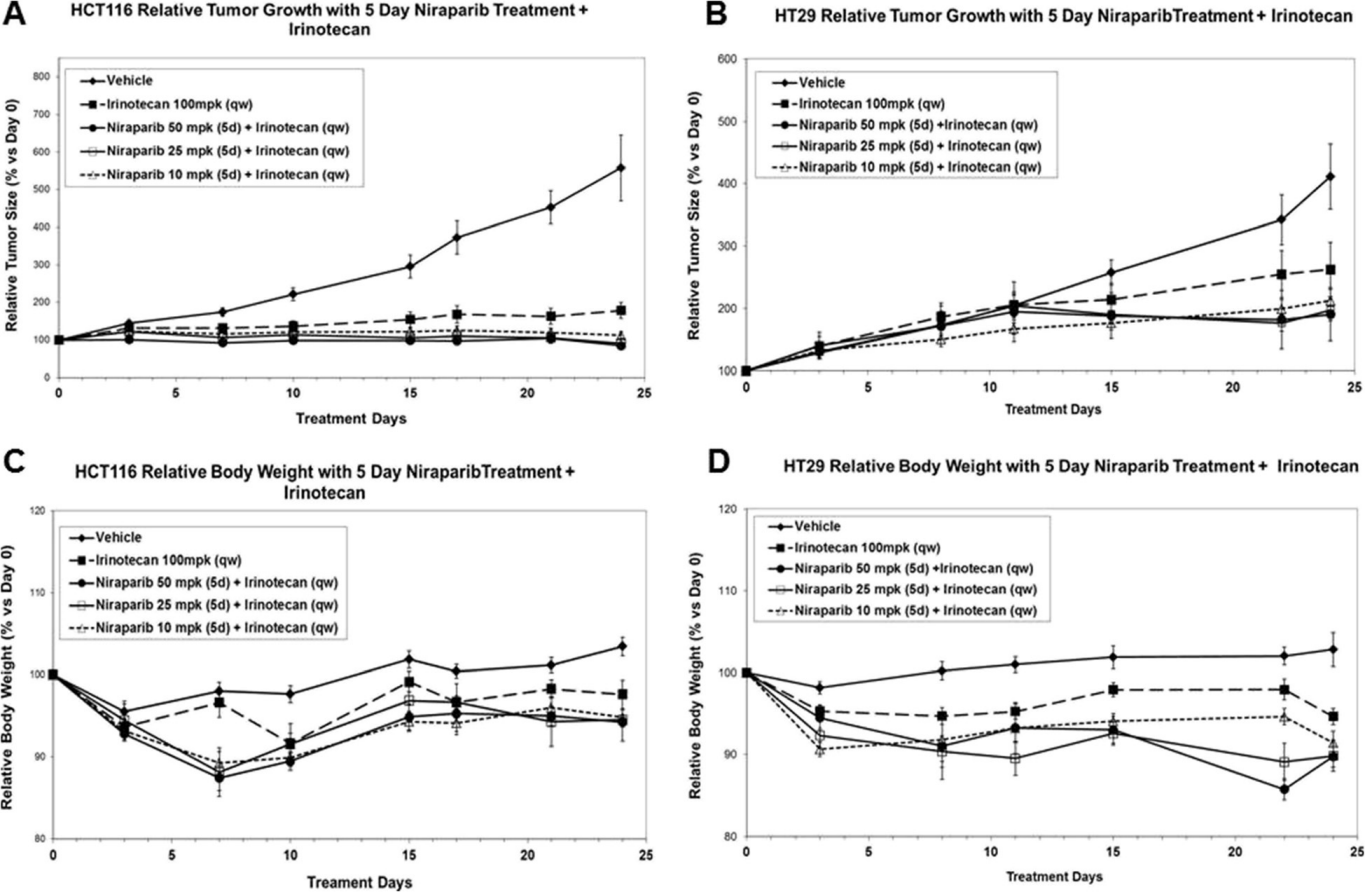
**Niraparib in combination with irinotecan is more efficacious than irinotecan alone in the HCT16 (MSI) CRC and HT29 (MSS) models.** (Data from this figure is from the same experiment as in Figure 4, but has been separated for ease of viewing.) *In vivo* efficacy of niraparib in combination with irinotecan in the **A**. HCT116 (MSI) and **B**. HT29 (MSS) xenograft models. Niraparib was dosed at 50, 25, or 10 mpk p.o. q.d., for 5 days a week (2 days off) and irinotecan was administered at 100 mpk i.p., on the 3rd day of every week for 4 weeks. Relative tumor size (% versus Day 0) is shown with SEM in error bars. **C**. and **D**. The 5-day dosing regimen was tolerated with 15% or less body weight loss in all combinations tested. Relative body weight (% versus Day 0) is shown. For each group, n = 7-10.

In the same studies, mice were also treated with niraparib (at 10, 25, and 50 mpk) in combination with irinotecan (100 mpk; qwk) to determine if niraparib could enhance irinotecan efficacy, and to determine if the combination would be tolerated. Niraparib significantly enhanced irinotecan efficacy at the 25 and 50 mpk combination dosing regimens in the HCT116 model, but not in the 10 mpk combination dosing regimen. The average tumor volume at day 28 in the Irinotecan group was 225 mm^3^, in the 50 mpk combination group was 120 mm^3^, the 25 mpk combination group was 101 mm^3^ and in the 10 mpk combination group was 166 mm^3^. Significance was determined using a one-tailed homoscedastic T test and p = 0.01, 0.02 and 0.06 for the 50 mpk, 25 mpk and 10 mpk combination groups as compared to irinotecan alone at day 28. In the HT29 model, the average tumor volumes for the combination groups were smaller at 24 days as compared to irinotecan, but these values were not statistically significant. The average tumor volume at day 24 in the Irinotecan group was 542 mm^3^, in the 50 mpk combination group was 363 mm^3^, the 25 mpk combination group was 438 mm^3^ and in the 10 mpk combination group was 448 mm^3^. Significance was again determined using a one-tailed homoscedastic T test and p = 0.09, 0.15 and 0.18 for the 50 mpk, 25 mpk and 10 mpk combination groups as compared to irinotecan alone at day 24. 15% or less body weight loss was observed throughout the duration of the studies (Figures 4C, 4D, 5C and 5D).

**Figure 6:**
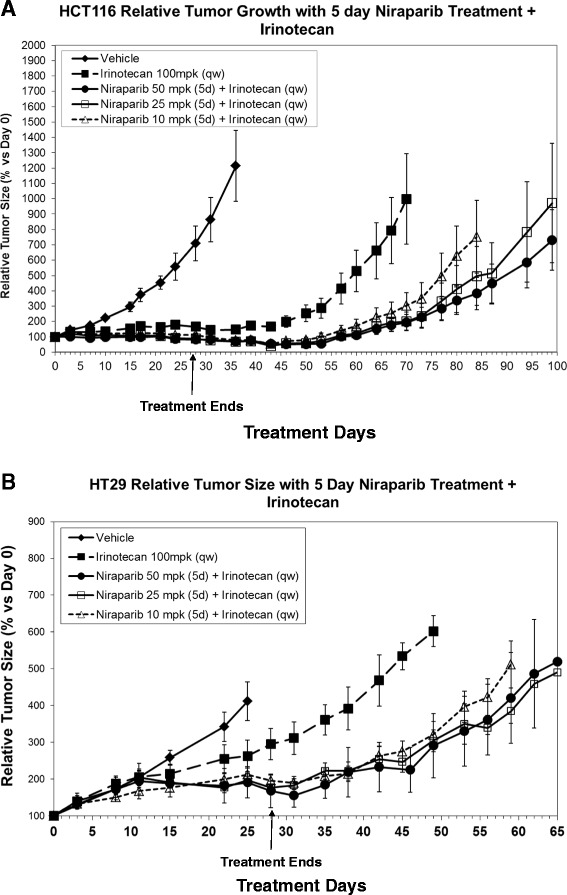
**Tumor growth delay is extended signficantly in niraparib + irinotecan combination treatment groups as compared to treatment with irinotecan alone.** (Data from this figure is from the same experiment as in Figures 4 and 5, but has been separated for ease of viewing). Tumor growth delay and relapse after the withdrawal of niraparib and irinotecan treatment in the **(A)** HCT116 (MSI) and **(B)** HT29 xenograft models. Niraparib was dosed at 50, 25, or 10 mpk p.o. q.d., for 5 days and irinotecan was administered at 100 mpk i.p. on the 3rd day of every week for 4 weeks. After the 4^th^ week of dosing of both drugs, treatment was stopped and tumor growth was monitored bi-weekly until the average tumor volume for each group reached to an end-point of 1000 mm^3^. Relative tumor size (% versus Day 0) is shown with SEM in error bars. For each group, n = 7-10. **(A)** The average tumor volume for the 50, 25, and 10 mpk niraparib + irinotecan combination groups in the HCT116 model were significantly different from the irinotecan single agent group at day 70 when the average tumor size in the irinotecan single agent group reached the end-point of 1000 mm^3^ with P-values of .02, .02, and .03, respectively. (P value generated using a homoscedastic Student’s t-test.) **(B)** The average tumor volume for the 50, 25, and 10 mpk niraparib + irinotecan combination groups in the HT29 model were significantly different from the irinotecan single agent group at day 49 when the average tumor size in the irinotecan single agent group reached the end-point of 1000 mm^3^ with P-values of .05, .03, and .03, respectively. (P value generated using a homoscedastic Student’s t-test).

In addition to assessing tumor growth inhibition during the 4 weeks with niraparib and irinotecan combination treatment, we also investigated tumor growth delay and relapse in the same studies after the withdrawal of treatments. The HCT116 and HT29 tumors treated with irinotecan alone relapsed sooner than tumors treated with niraparib in combination with irinotecan at all 3 niraparib doses tested (Figure 6). In the HCT116 study, the 50 mpk niraparib + irinotecan combination treatment group reached the 1000 mm^3^ endpoint at day 99, whereas the irinotecan alone treatment group reached the 1000 mm^3^ endpoint at day 70, demonstrating a 29-day tumor growth delay. Additionally, the average tumor volume for the 50, 25, and 10 mpk niraparib + irinotecan combination groups in the HCT116 model were significantly different from the irinotecan single agent group at day 70 when the average tumor size in the irinotecan single agent group reached the end-point of 1000 mm^3^.(P = .02, .02, and .03, respectively. P values generated using a homoscedastic Student’s t-test.) (Figure 6A). Likewise, in the HT29 study, the 50 mpk niraparib + irinotecan combination treatment group reached the 1000 mm^3^ end-point at day 65, whereas the irinotecan only treatment group reached the end-point at day 49, demonstrating a 16-day tumor growth delay. The average tumor volume for the 50, 25, and 10 mpk niraparib + irinotecan combination groups in the HT29 model were significantly different from the irinotecan single agent group at day 49 when the average tumor size in the irinotecan single agent group reached the end-point of 1000 mm^3^. (P-values of .05, .03, and .03, respectively. P value generated using a homoscedastic Student’s t-test) (Figure 6B). Collectively, our *in vitro* and *in vivo* data demonstrate that combination of niraparib with irinotecan is efficacious in both MSI and MSS settings.

## Discussion

To determine if MSI is associated with increased sensitivity to the PARP inhibitor niraparib and to determine if niraparib potentiates the anti-proliferative effects of irinotecan, the efficacy of niraparib and irinotecan, both alone and in combination, was assessed in multiple MSI and MSS CRC models *in vitro* and *in vivo*. The studies detailed in this paper demonstrate that CRC MSI cell lines are not more sensitive than CRC MSS cell lines to niraparib, and that combination of niraparib with irinotecan enhances the efficacy of irinotecan in both MSI and MSS CRC cell lines *in vitro* and *in vivo*.

We have demonstrated that MSI CRC cell lines have reduced levels of Mre11, a protein involved in the repair of DSBs, as compared to MSS cell lines, but that they are not significantly more sensitive to niraparib monotherapy than MSS cell lines (Figures 1 and 2A). Even the cell lines that had little or no detectable levels of Mre11 (RKO, LS411N, HCT116; Figure 1) did not respond to niraparib monotherapy in the EC50 ranges that BRCA deficient cell lines do (EC50s ≥ 1000 nM for MSI CRC cell lines and EC50s ≤ 100 nM for BRCA1/2 mutant cell lines using the same assay conditions) (Figure 2A and Table 1; [3]). This data demonstrates that reduction of Mre11 levels to the degree that they are reduced in the context of MSI, is not sufficient to induce sensitivity to PARP inhibition. The notion that Mre11 deficiency is fundamentally different from BRCA1/2 deficiency is supported by the observation that germline inactivation of Mre11 does not result in a cancer predisposition syndrome, whereas inactivation of BRCA1/2 does.

Our results differ from those published for the PARP inhibitor ABT-888 by Vilar et al., in 2011 and the PARP inhibitor BMN673 by Gaymes et al., in 2013 [28,29]. Vilar et al. reported that Mre11 deficiency increases sensitivity to PARP inhibition in MSI CRC. Discrepancies in our results are likely due to the size of the cell line panels that were used. The panel used in our study was larger than what Vilar et al., used. All of the MSI cell lines that were assayed in the Vilar manuscript were assayed in our panel with an additional 4 MSI lines. In addition, 7 out of the 9 of the MSS cell lines that were assayed in their panel were assayed in our panel with an additional 6 MSS lines. When statistical analysis is performed on our 7 day niraparib EC50 data using only the cell lines that were used in the Vilar study, we demonstrate that MSI cell lines are significantly more sensitive to niraparib than MSS cell lines (p = 0.02). This data highlights the need to include large numbers of cell lines to evaluate biomarker hypotheses. Additionally, Vilar et al., describe the differences between MSI and MSS cell lines using 10 μM (and 50 μM) of ABT-888, which is more than 10 times the EC50 reported for ABT-888 in the BRCA mutant context [31]. Notably, the concentrations of compound used in those studies is unlikely a therapeutically relevant dose [32].

Gaymes et al., reported that MSI induced mutations in DNA repair genes confer hypersensitivity to the PARP inhibitor BMN673 in myeloid malignancies [29]. These authors were studying cell lines of myeloid lineage and we did not assay any of the same cell lines in our studies. Discrepancies in our results could stem from different cell line panels, the effects of MSI in other tissue types, or a difference in assay platform.

We have also confirmed previous reports that MSI CRC cell lines are more sensitive to irinotecan (SN-38) monotherapy than MSS CRC cell lines in a large cell line panel. (Figure 2B and Table 1) [18]. The average 7 day EC50 for SN-38 in MSI cell was 2.1 nM and for MSS cells was 10.0 nM. However, 6/13 MSS cell lines tested had EC50s less than 5nM (Figure 2B and Table 1). The data in Figure 2B. demonstrates that there are SN-38 sensitive and SN-38 insensitive subpopulations of MSS cell lines. Some of the MSS cell lines are quite sensitive to SN-38 and we have demonstrated that if a cell line responds to SN-38 monotherapy then niraparib will potentiate that effect, in both MSI and MSS cell lines (Table 1). We have also demonstrated that combination of niraparib with SN-38 results in additive to synergistic inhibition of cell proliferation in both MSI and MSS CRC cell lines *in vitro* (Figure 3). In addition, we demonstrate that while the doses of niraparib used in this study are not as efficacious as irinotecan in monotherapy, the combination of low doses of niraparib and irinotecan results in greater tumor growth inhibition in MSI and MSS tumor models *in vivo* (Figure 4.) In tumor growth delay and relapse studies, tumors treated with irinotecan alone relapsed earlier than tumors treated niraparib and irinotecan combination at all three niraparib doses tested (50, 25, 10 mpk) in both MSI and MSS CRC xenograft models.

Our *in vitro* and *in vivo* preclinical results demonstrate that in CRC cell lines, MSI does not render cells more sensitive to niraparib, but that combination of niraparib with irinotecan enhances the efficacy of irinotecan in both MSI and MSS CRC cell lines. Our data suggests that both MSI and MSS patient populations will benefit from the combination of niraparib and irinotecan.
